# Civil-military cooperation to contain COVID-19 epidemic in Israel- Lesson learned

**DOI:** 10.1093/eurpub/ckac131.059

**Published:** 2022-10-25

**Authors:** Z Mor, N Davidi, S Alroy Preis

**Affiliations:** 1 Public Health, Ministry of Health, Tel Aviv, Israel; 2 School of Health Sciences, Ashkelon Academic College, Ashkelon, Israel; 3 Home Front Defence, Israeli Defence Forces, Ramla, Israel

## Abstract

**Issue:**

Public Health Services (PHS) has a major role in controlling COVID-19. As the epidemic propagated, and due to limited resources, PHS in Israel reached it capacity to contain the outbreak. Following a political decision in June 2020, the Home Front Command (HFC) of the Israeli Army was assigned to integrate in operating the National epidemiological efforts, while the PHS remained responsible for policy, setting guidelines and supervision.

**Problem:**

The civilian PHS and the military HFC had to establish cooperation, institute new hierarchical structure and divide the responsibility, despite the differences in organizational cultures, financial and human resources. Additionally, inter and intra organizational interests had to be resolved.

**Results:**

Formal and informal efforts were needed to bridge between the two organizations, while utilizing the comparative advantage of each agency. PHS has experience in controlling outbreaks, well-established intra-organizational communication, high professional identity, commitment and familiarity with different populations in Israel. HFC is a flexible, creative, learning and fast-responding organ, experienced in controlling emergencies and has well-established chains of command. HFC is supported by IT and intelligence officers. Organizational disadvantages had to be resolved. PHS is deficient in resources, has limited capacity in operating staff during irregular hours, and is obliged to share the health leadership and authority with the army. HFC has to confront high rotation of its manpower, difficulty in succumbing to non-military guidelines and regulations and possible mistrust between the army and special populations, such as Arabs or ultra-religious Jews.

**Lessons:**

Civil-military epidemiological cooperation can boost the National response in containing epidemics. Policymakers from each agency should use leadership skills to encourage integration while being sensitive to the needs and expectations of all participants.

**Key messages:**

• Civil–military epidemiological cooperation boosts the National response in containing epidemic. Mutual organizational sensitivity is crucial for constructive integration by leaders from each agency.

• Civil–military epidemiological cooperation boosts the National response in containing epidemic. Mutual organizational sensitivity is crucial for constructive integration by leaders from each agency.

